# Design and Test of a Soft Plantar Force Measurement System for Gait Detection

**DOI:** 10.3390/s121216628

**Published:** 2012-12-03

**Authors:** Xuefeng Zhang, Yulong Zhao, Zhengyong Duan, Yan Liu

**Affiliations:** State Key Laboratory for Manufacturing Systems Engineering, Xi’an Jiaotong University, No. 28, Xianning West Road, Xi’an, Shaanxi 710049, China; E-Mails: bssair@163.com (X.Z.); dzyai@163.com (Z.D.); ZTlover@stu.xjtu.edu.cn (Y.L.)

**Keywords:** MEMS, plantar force, gait detection, flexible

## Abstract

This work describes a plantar force measurement system. The MEMS pressure sensor, as the key sensing element, is designed, fabricated and embedded into a flexible silicon oil-filled bladder made of silicon rubber to constitute a single sensing unit. A conditioning circuit is designed for signal processing and data acquisition. The characteristics of the plantar force sensing unit are investigated by both static and dynamic tests. A comparison of characteristics between the proposed plantar force sensing unit and a commercial flexible force sensor is presented. A practical experiment of plantar force measurement has been carried out to validate the system. The results demonstrate that the proposed measurement system has a potential for success in the application of plantar force measurement during normal gait.

## 1. Introduction

The plantar force is frequently measured as an important biomechanical parameter that can reflect the supporting situation and stability of the human gait. Potential applications of a plantar force measurement system include rehabilitation science [1–4], athletic sports [5,6], robotics technology [7], *etc.* Development of a simple, easily used, high-performance prototype for plantar force measurement has attracted extraordinary research interest.

Most current plantar force sensors are based on conductive polymers [8–12]. However, research shows that their performance with regards to repeatability, hysteresis, creep and linearity fail to meet the requirements of accurate plantar force measurement [12–14]. Various improved designs have been proposed to solve the above-mentioned problems. Tomizuka developed a smart shoe to monitor the human gait by embedding a commercial air pressure sensor in a shoe [3]. However, the complication of its compensation algorithm might restrict its popularity. Chedevergne *et al.* presented a plant force sensor formed by a metallic ring equipped with a strain gauge [15], but the proposed metallic element would introduce discomfort and alter the natural gait. New devices have sprung up with the development of new materials and technology. De Rossi *et al.* introduced a pressure sensitive device consisting of silicone covered opto-electronic pressure sensors and a high-frequency data processing system [16]. Shu *et al.* provided a design of a plantar pressure measurement system based on a fabric pressure sensing array [17]. Both of them offer new approaches for plantar pressure measurement. Karki *et al.* reported a piezoelectric polymer film sensor for plantar normal and shear stress measurements [18]. The signal conditioning of piezoelectric sensors is complicated, and the influence of a charge amplifier on the dynamic characteristics of a piezoelectric sensor should be analyzed carefully. Meantime, several flexible capacitive sensors have been proposed and tested [19–21], but the accuracy of the capacitive sensor is greatly affected by the external environment. Meanwhile, piezoresistivity has been widely used in the design of MEMS pressure sensors because of its simple signal processing, high sensitivity, and excellent linearity. Piezoresistive MEMS pressure sensors have been used in biomechanical applications by employing special packing structures and designs [12,22]. However, difficult processes were required to package pressure sensors in a sole. Meanwhile, the failure of pressure sensors when overloaded must be taken into account.

Considering the features mentioned above, a soft plantar force measurement system with a MEMS pressure sensor is proposed in this paper. By encapsulating a piezoresistive pressure sensor into a silicon oil-filled bladder made from silicon rubber, a sensing unit with good performance is expected. In addition, overload protection is anticipated due to the existence of silicon oil to transmit the pressure and limit the compression of the bladder. As piezoresistivity is utilized to realize the sensing unit, low cost and a simple signal process can be expected. To accurately measure the plantar force, three plantar force sensing units are placed in the sole, and the processing electrics and data acquisition circuit are combined to realize the plantar force measurement system. The proposed prototype has been applied in the practical plantar force measurement, and comparisons have been done to evaluate the effectiveness of the proposed measurement system.

## 2. Design of the Pressure Sensor

**Figure 1.**
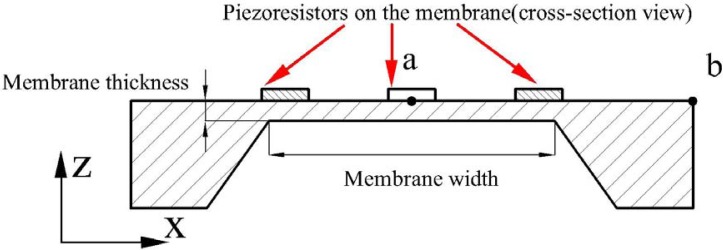
Conceptual design model of the proposed micro pressure sensor.

A MEMS pressure sensor is used as the key component in detecting the variation of the plantar force during walking. The sensor consists of a square diaphragm with four p-type piezoresistors located on the edges of diaphragm. A full Wheatstone bridge is integrated in the sensor chip to utilize the longitudinal and transversal piezoresistive effect of silicon. The conceptual design model of the pressure sensor is shown in Figure 1.

A square membrane is used as the sensitive structure for its higher induced stress when compared with a circular membrane. Theoretical model of the square membrane is established to specify the relationship of length, thickness and effective stress. According to the mechanics principle of the periphery fixed square diaphragm, the maximum deflection at the central point a can be expressed as:

(1) $$w_{\text{max}}=0.0151\frac{pa^{4}}{Eh^{3}}(1-v^{2})$$

where *a* and *h* denote the length and thickness of the diaphragm and *p* is the applied pressure. *E* and *v* are the Young’s modulus and the Poisson ratio of silicon, respectively. The maximum stress occurs at the middle of four edges and is given by:

(2) $${\left|\sigma \right|}_{\text{max}}=0.308\frac{pa^{2}}{h^{2}}$$

The corresponding strain is:

(3) $$\varepsilon =\frac{1-v}{E}\sigma$$

**Figure 2.**
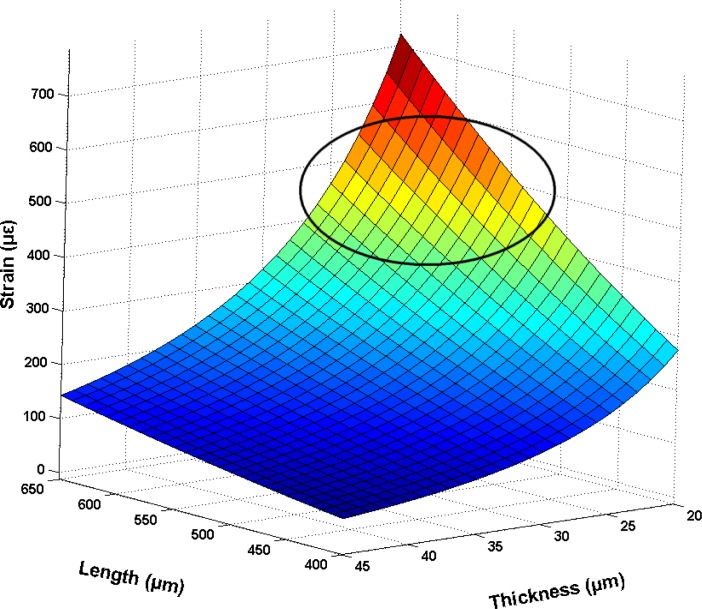
Calculated strain *versus* the diagram size and thickness.

Numerical analysis by Matlab is performed to identify the effective range of parameters. The pressure applied on the sole is up to 400 kPa during walking [21], therefore the load is set as 400 kPa, and the obtained results are shown in Figure 2. Based on the numerical analysis, a set of data of diaphragm thickness and length (marked by the circle) are chosen for further optimization.

**Figure 3.**
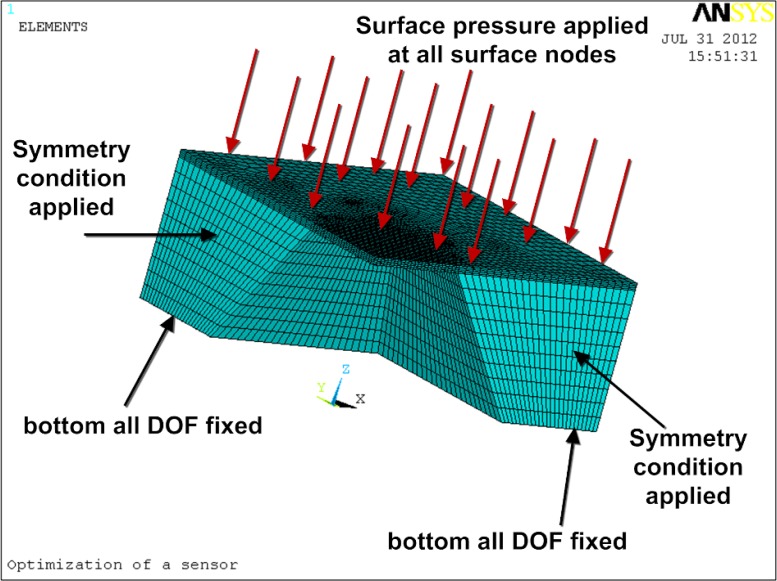
Meshed ANSYS model with the applied boundary conditions.

FEM optimal design based on ANSYS is performed to finally specify the feasible parameters. The determined structural parameters can guarantee that the pressure sensor have the highest attainable sensitivity and stability at the same time. One fourth of the chip is modeled and simulated considering its complete symmetry. Figure 3 shows the finite element model with the constrain conditions and applied pressure.

**Figure 4.**
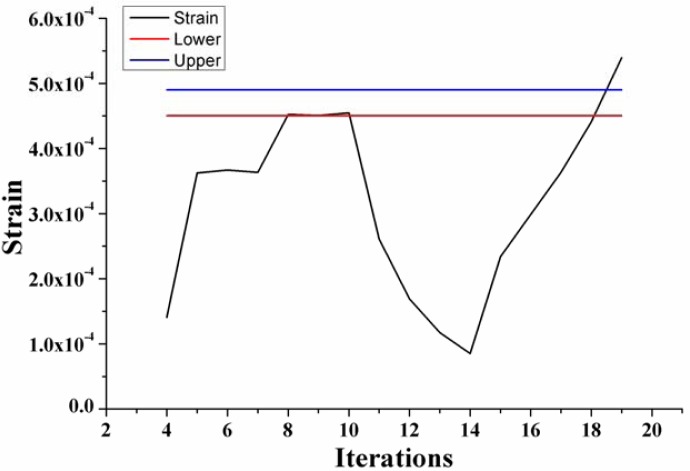
State variable change curve with the iterations.

In the optimal design, the thickness and length of diaphragm are the design variables, strain is the state variable and minimum volume is the objective [23]. According to the numerical analysis results and process capacity, the thickness is considered in a range of 20–45 μm and length is in a range of 400–600 μm, respectively. The strain is confined in the range of (450–490) × 10^−6^ to assure a sensor output with high linearization [24]. Figure 4 shows the state variable change curve with the iterations.

**Figure 5.**
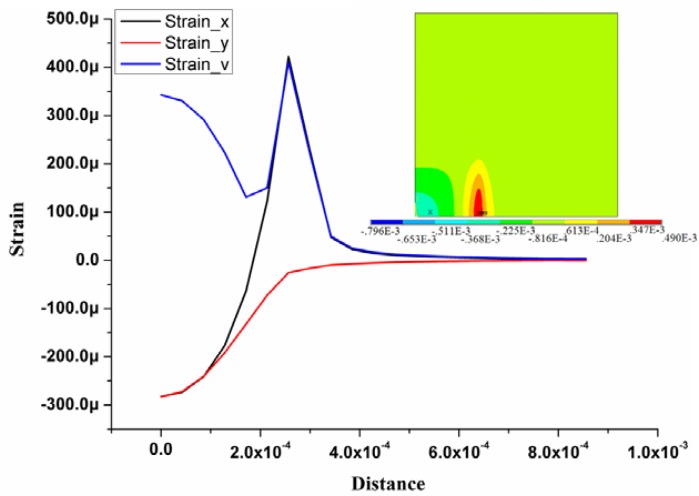
Analytical results of the quarter model of the sensor chip based on ANSYS.

From the optimal result, the combination of diaphragm length 553 μm and thickness 20 μm is an optimal solution for the sensor design, which can balance the reliability and the sensitivity. The FEM simulation results of the pressure sensor chip with defined dimensions are shown in Figure 5, which shows the strain distribution on the top surface of the sensor chip and the strain distribution curve along the defined path. The path starts from the sensor chip center (point a) to the edge (point b) along the x-axis, as demonstrated in Figure 1. Strain_v, strain_x and strain_y represent the von-Mises, transverse, and longitudinal strain results, respectively. The maximum strain difference is located at the edge of the membrane where the resistors are implanted.

## 3. Realization of the Measurement System Prototype

### 3.1. Fabrication of the Pressure Sensor

**Figure 6.**
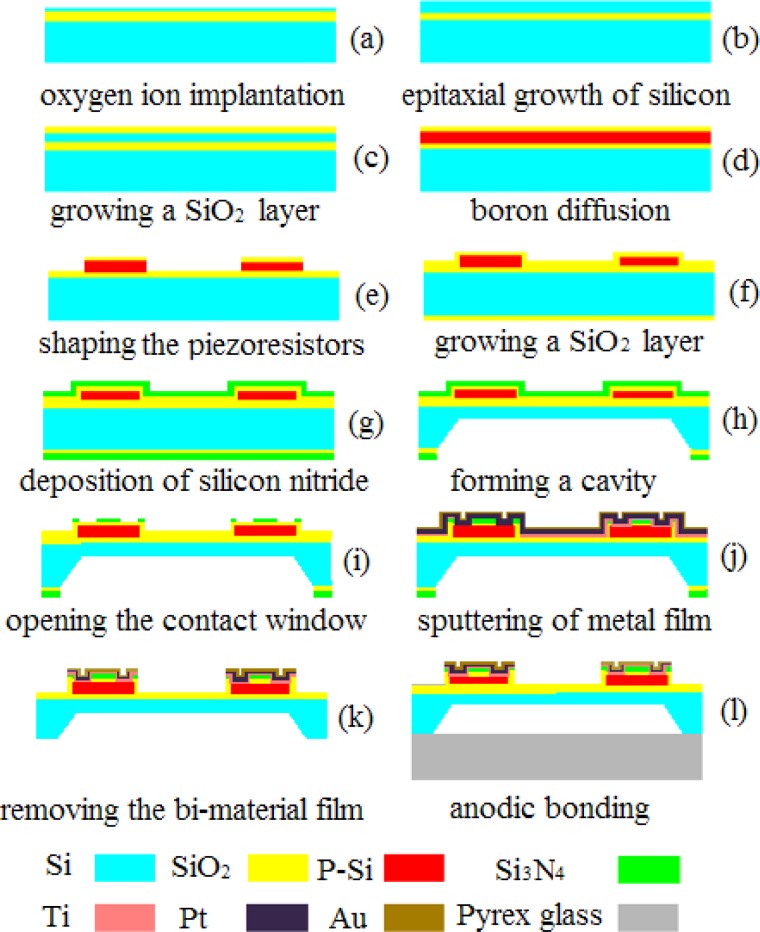
Fabrication process steps of the micro pressure sensor.

The pressure sensors were fabricated by silicon micromachining. Silicon on insulator (SOI) technology is used to fabricate the designed pressure sensor, to reduce parasitic device capacitance, thereby improving performance [25]. A 400 μm, double side polished, n-type, (100) oriented silicon wafer was used as the starting material. The fabrication process steps are illustrated in Figure 6.

**Figure 7.**
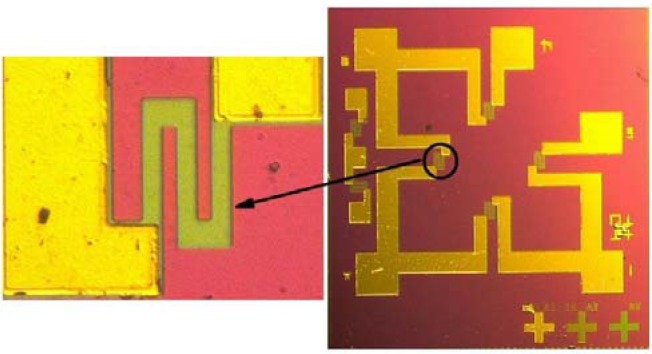
Photograph of a fabricated micro pressure sensor chip.

A total of four masks were necessary, for the fabrication of piezoresistors, the etching of back cavity, the fairlead and the metal wire. The fabrication sequences started with creating a buried SiO_2_ layer by oxygen ion implantation (a), epitaxial growth of silicon using LPCVD (b), growing a SiO_2_ layer using thermal oxidation (c), followed by boron diffusion for resistors (d), preliminary shaping the piezoresistors by plasma etching (e), growing a SiO_2_ layer by thermal oxidation (f), depositing a silicon nitride layer (g), forming a cavity on the backside by wet etching technique (h), creating the bond pads and conductors (i,j,k), and ended with attachment of Pyrex glass to the silicon substrate by anodic bonding (l). With respect to the boron diffusion, light impurity concentration was controlled at about 5 × 10^18^ cm^−3^ and thus the nominal sheet resistance approximated to 200 Ω. By applying such processes, a micro pressure sensor chip was fabricated as shown in Figure 7, with one of the piezoresistors in an enlarged view.

**Figure 8.**
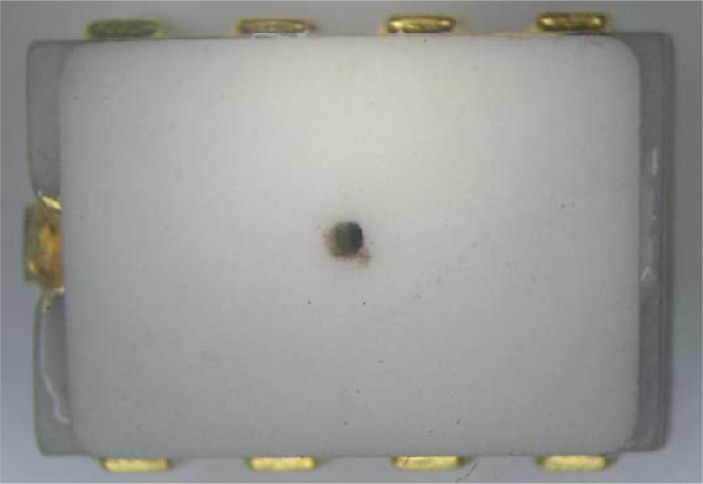
Photograph of a micro pressure sensor after package.

To prepare for subsequent applications, a ceramic dual-in-line package was designed to provide the sensor chip with mechanical support, power and signal paths, environmental protection, and/or interface protection [26]. A strong epoxy adhesive was used to mount the chip into the package, and the pads were bonded to the pins correspondingly. A lid with an air inlet was glued on the package to finish the package [27,28]. A packaged pressure sensor is shown in Figure 8.

### 3.2. Assembling of the Sensing Unit

The plantar force sensing unit was designed by encasing the fabricated pressure sensor into a flexible spherical bladder made of silicon rubber. Silicon rubber is used because it exhibits good stability, self-healing and high breakdown field strength [29]. The spherical bladder was designed with flanging and fabricated by injection molding process. This was helpful to improve the operating reliability of the sensing unit by increasing the adhesive area and strength.

To protect the pressure sensor and increase the measuring range of the sensing unit, silicon oil is filled into the silicon rubber bladder as pressure transfer medium, because it has some advantages over other materials such as small compressibility, high purity and high electric resistivity [30]. This proposal provides a flexible interface between the human tissue and the pressure sensor while maintaining high measurement accuracy.

**Figure 9.**
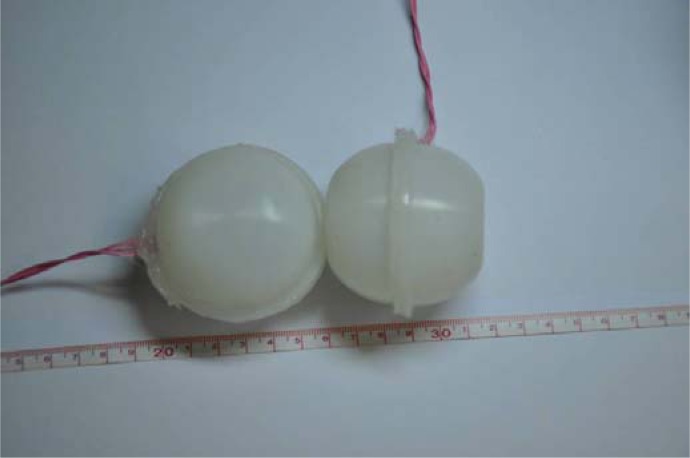
Photograph of plantar force sensing unit.

In the plantar sensing unit, electrical connection for power supply and signal transmission is required without any leakage of silicon oil. A lead slot is carefully designed on the edge of the spherical bladder to solve this problem. A special wire coated with rubber is used to transmit the power and signal, for it will provide good adhesive properties matching with the silicon rubber. Then, the bladder is bonded and sealed by silicon rubber adhesive. Prototypes of the plantar force sensing units are shown in Figure 9.

### 3.3 Design of the Measurement System

**Figure 10.**
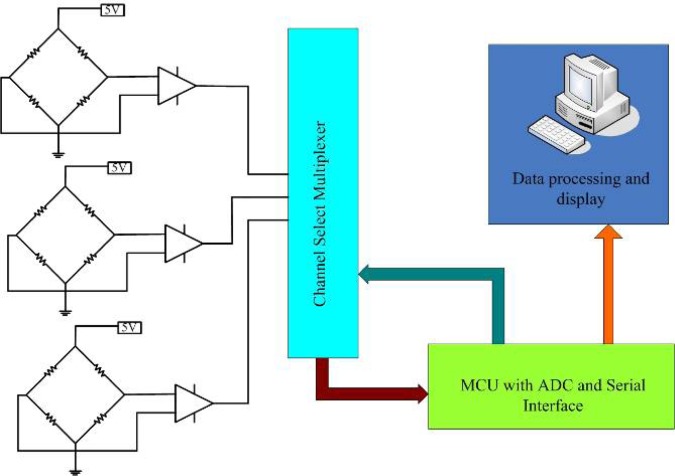
Schematic diagram of the conditioning circuit.

A conditioning circuit is designed to acquire, amplify and store the plantar force signal. The schematic diagram of the conditioning circuit is given in Figure 10. Outputs of the three pressure sensors are amplified separately, and then a multiplexer is used to switch channel to an analog digital conversion device. Finally, the digital signal of plantar force is sent to a computer by the serial port for display and storage.

**Figure 11.**
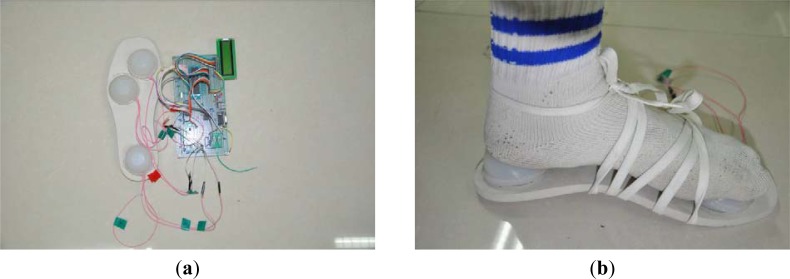
Pictures of the plantar force measurement test. (**a**) The designed plantar force measurement system; (**b**) The scene of the plantar force measurement.

Three plantar force sensing units and the conditioning circuit are assembled into a sole to build up the plantar force measurement system. Three units are placed under the toe, sole and heel respectively according to the anatomy of the human foot. The designed plantar force measurement system is shown in Figure 11(a). Figure 11(b) shows the simple setup of the plantar force measurement.

## 4. Experimental Setup and Results

**Figure 12.**
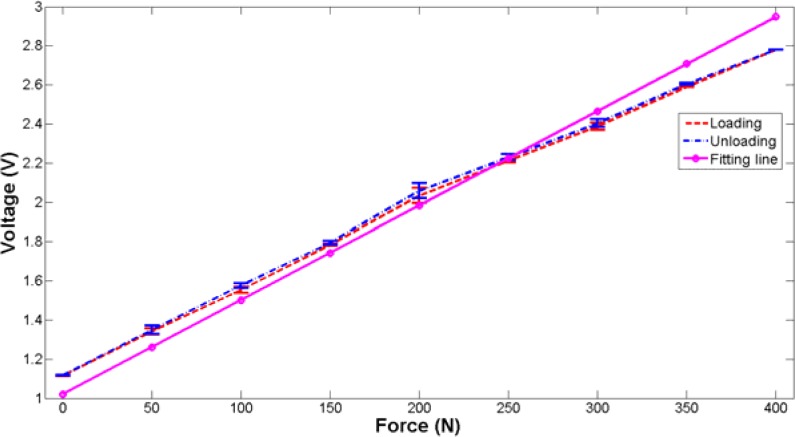
The static performance of the designed plantar force sensor.

**Figure 13.**
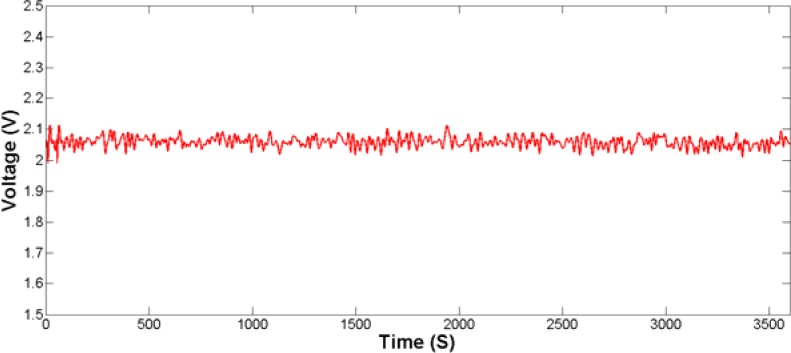
The creep of the designed plantar force sensor for 1 h.

Static and dynamic tests are performed to evaluate the performance of the designed plantar force measurement system. The static characteristics of the sensing unit were investigated by an electronic universal material testing machine (UTM6104, Shenzhen Suns Technology Stock Co. Ltd., Shenzhen, China). The sensing unit was placed on the worktable of the testing machine. The applied force rose from 0 N to 400 N and decreased to 0 N in one test. The maximum applied force was determined according to the previous test parameters [31]. Tests were repeated four times and the average value of output voltage was calculated. The experimental data is fitted with the Least Square Method. Figure 12 shows the fitting results of the output voltage as a function of applied force with standard deviation denoted. It can be obviously seen that the fabricated plantar force sensing unit exhibits an average sensitivity (the slope) of 4.17 mV/N. Hysteresis is 1.26%, which is mainly due to the viscoelasticity of the silicon rubber. The linearity and repeatability are 8.71% and 0.71%, respectively. The nonlinearity is mainly caused by the viscous materials used in the sensing unit. To investigate the creep of the sensing unit, a constant force of 200 N is exerted. The output of the sensing unit is recorded over an hour to evaluate the creep as shown in Figure 13. As to the creep value, the calculated maximum error over an hour is about 6.2%.

**Figure 14.**
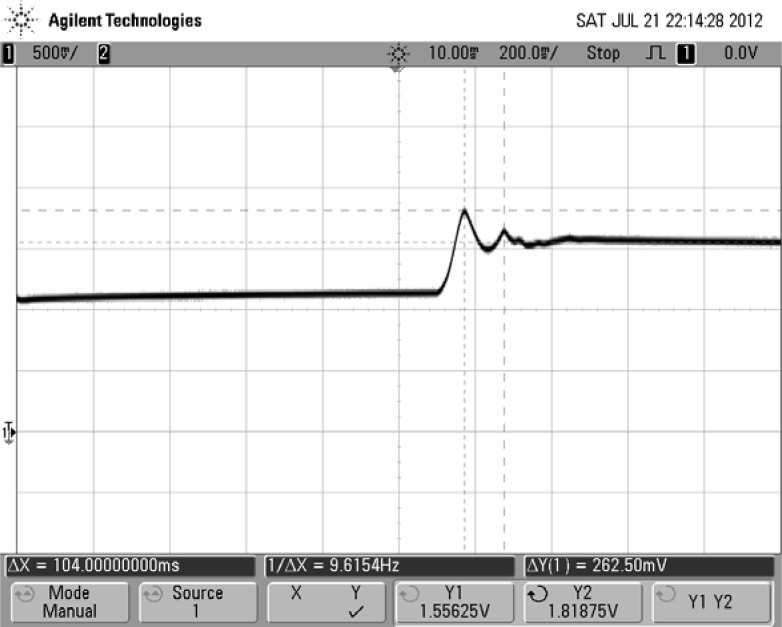
Response of the sensor to a step force.

The dynamic test is implemented by imposing a step force that is generated by rapidly loading a 15 kg weight on the bladder. Figure 14 shows the dynamic response of the sensing unit to the step force. The peak time and oscillation period of the signal are obtained by observing the curve. The response frequency of the designed sensing unit is about 9.6 Hz. This low frequency may be mainly induced by low stiffness and high internal damping of the materials used in the prototype. However, this frequency range is enough for normal gait detection based on the results reported in [13].

Table 1 compares the characteristics of the designed plantar force sensing unit with one commercial flexible force sensor. It is obvious that the plantar force sensor presented in this paper has better performances.

**Figure 15.**
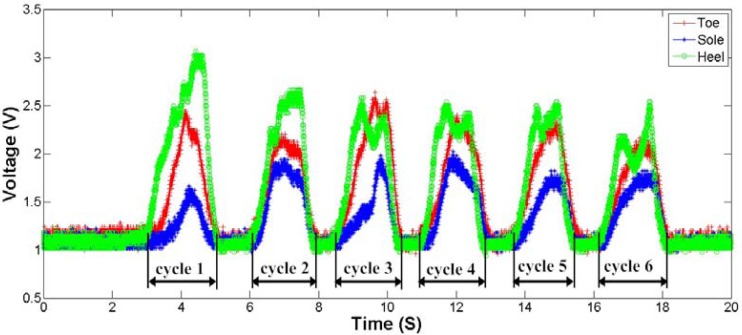
Voltage time waveform obtained from the plantar force measurement.

Finally, a simple practical experiment of plantar force measurement is carried out in order to validate the designed system. The plantar force measurement sole is placed and fastened under the foot of a subject. The subject repeats the mark time action considering the motion is restricted by the signal cable. The actions were repeated randomly for six times in this measurement. Figure 15 shows the output curves of the three sensing units. The maximum plantar force appears beneath the heel, and minimum force locates under the sole. By analysis the output signal of three sensing units, we can collect the useful information of the plantar force without difficulty. These data can be further processed and used for clinical purposes, such as gait detection and so on.

## 5. Conclusions

This paper presents the development of a plantar force measurement system prototype for human gait detection. The proposed prototype consists of three plantar force sensing units and a conditioning circuit for data processing and acquisition. The sensing unit uses a MEMS-based pressure sensor as the sensing element and a silicon oil-filled bladder as the pressure transmission medium. The dimensions of the pressure sensor chip are specified by theoretical modeling and FEM size optimization. The experimental results show that the sensing unit features high repeatability (≤0.71%), low hysteresis (≤1.26%), small creep (≤6.2%), and acceptable linearity (≤8.71%). The measured dynamic response frequency is about 9.6 Hz, which is enough for normal human gait detection. Practical measurements show that the proposed system can reflect the foot motion in mark time action. The developed plantar force measurement system prototype combines the advantages of MEMS-Based sensors and flexible interface of rubber bladder, confirming the feasibility of embedding a micro pressure sensor chip into a flexible bladder to detect the human gait. Future work will be focused on the package optimization to improve the structural compactness, and the incorporation into a wireless sensor network for better and wider practical applications has also been be put on the agenda.

## Figures and Tables

**Table 1.** Comparisons with a commercial sensor.

**Device**	**Linearity**	**Hysteresis**	**Repeatability**	**Frequency**	**Creep**
Flexiforce sensor [32]	≤±10%	≤15%	≤5%	Not available	<15%
This works	8.71%	1.26%	0.71%	9.6 Hz	6.2%
